# Sequestration of trivalent americium and lanthanide nitrates with bis-lactam-1,10-phenanthroline ligand in a hydrocarbon solvent†

**DOI:** 10.1039/c9ra06115k

**Published:** 2019-08-23

**Authors:** Yana Karslyan, Frederick V. Sloop, Laetitia H. Delmau, Bruce A. Moyer, Ilja Popovs, Alena Paulenova, Santa Jansone-Popova

**Affiliations:** Laboratory of Transuranic Elements, Department of Chemistry, Oregon State University Corvallis OR 97331 USA; Chemical Sciences Division, Oak Ridge National Laboratory Oak Ridge TN 37831 USA jansonepopos@ornl.gov; Isotope and Fuel Cycle Technology Division, Oak Ridge National Laboratory Oak Ridge TN 37831 USA

## Abstract

Efficient separation of minor actinides and lanthanides from used nuclear fuel could potentially lead to the development of sustainable nuclear fuel cycles. Herein, we report an in-depth study on selectivity and speciation in the extraction of the trivalent minor actinide Am and rare earth metal ions with a pre-organized phenanthroline-based ligand in a hydrocarbon solvent system relevant to nuclear fuel reprocessing. The 1 : 1 and 2 : 1 ligand-to-metal complexes dominate the speciation in the organic solvent over a range of ligand-to-metal concentrations, as evidenced by experimental data and supported by modeling.

## Abstract 2

Efficient separation of trivalent americium and light lanthanides from aqueous acidic medium into hydrocarbon solvent using BLPhen extractant.

## Graphical abstract

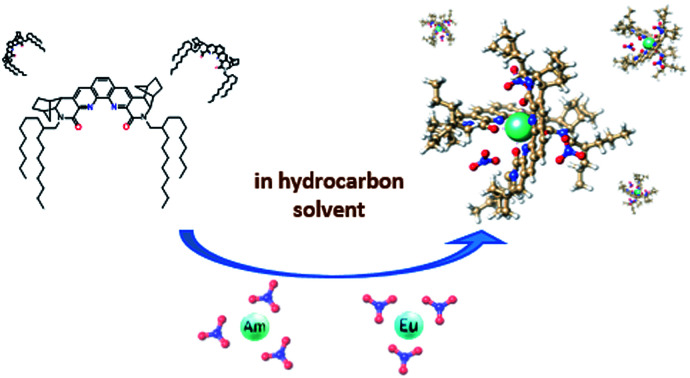

## Introduction

The minor actinides (MA) americium, curium, and neptunium have high radiotoxicities and americium is the main heat contributor present in used nuclear fuel after the fission products ^137^Cs and ^90^Sr have decayed in the first 300 years. Lanthanides (Ln) on the other hand, being neutron absorbers, hamper the transmutation of actinides and must be removed for MA recycling in mixed oxide fuel. The selective partitioning and transmutation of minor actinides following the Plutonium Uranium Redox Extraction (PUREX) process will therefore decrease the heat generation and long-term radiotoxicity of the used nuclear fuel, enabling safer long-term storage.^1^ A single process of MA separation is desired ideally; however, due to similar chemical and physical properties of MA and Ln, it is rather a challenging task to realize. Alternatively, a coextraction of MA and Ln by a mixture of neutral and acidic extractants in organic solvent, followed by selective back-extraction of MA using aqueous aminopolycarboxylate complexants, has been recognized as a robust and effective process to achieve mutual separation.^2^ The selective isolation of f-elements is dictated by the modes of interaction with the ligands, and the choice of media used in liquid–liquid separation system. According to hard and soft Lewis acid and base theory, the covalency in complexes between 5f element and ligand is greater than in 4f element complexes and is strongly dependent on the nature of ligand.^3^ The number of potential coordination sites and the level of preorganization in ligand are important factors that can influence the affinity and selectivity towards particular f-block metal ions. In comparison to conformationally flexible chelators, preorganized multidentate ligands show improved affinity for f-elements, owing to convergent positioning of the donor atoms at the metal binding site.^4–8^ Performance of new unsaturated heterocyclic extractants is usually evaluated in polar chlorinated or aromatic solvents. This is in large part due to limited solubility in non-polar aliphatic diluents such as *n*-dodecane, Isopar, and kerosene, despite their low toxicity, low viscosity, high flash point, and unsuitability for an industrial-scale extractions.^9^

Phenanthroline-based ligands have received increased amount of attention due to their excellent ability to separate trivalent actinides and lanthanides.^6,10^ Previously, we have demonstrated application of the preorganized bis-lactam-1,10-phenanthroline (BLPhen) ligand in efficient separation of americium from europium^7^ and showing preferential selectivity for light-*versus*-heavy trivalent lanthanides.^8^ However, unsatisfactory solubility of BLPhen ligand 1 in non-polar diluents required the use of 1,2-dichloroethane-based extraction system. This, in turn, resulted in complete extraction of both Am and Eu from aqueous solution due to abnormally high binding affinity of this ligand, hampering the elucidation of both selectivity and speciation in the organic phase. Herein, we present a study that addresses these shortcomings by using a new branched-chain ligand from this family, BLPhen ligand 2. This ligand has a markedly better solubility in hydrocarbon diluent, therefore allowing its performance to be evaluated with the emphasis on speciation of f-element complexes with BLPhen ligand in more appropriate hydrocarbon solvent, which is significantly less toxic and is not a suspected carcinogen.

## Results and discussion

**Fig. 1:**
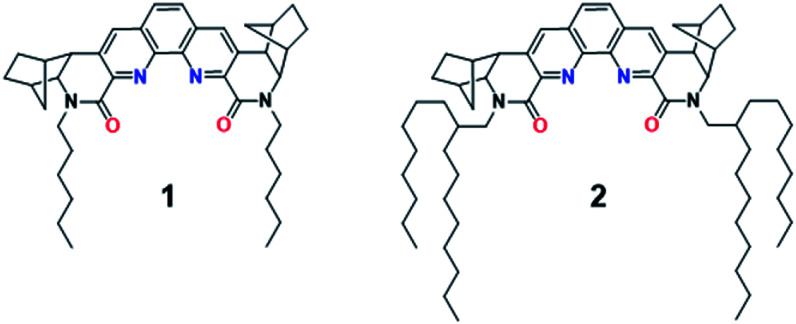
Chemical structure of known BLPhen ligand 1 and new BLPhen ligand 2.

The phenanthroline-based ligands usually have very limited solubility in hydrocarbon solvents due to increased polarity and likely π–π stacking due to the presence of extended π-surfaces. The solubility in non-polar diluents can be significantly improved by introducing long and branched alkyl substituents that disrupt the π–π stacking. Indeed, the introduction of a 2-octyldecyl group on the two lactam nitrogens resulted in ligand 2, shown in Fig. 1, which in comparison to bis-lactam-1,10-phenanthroline ligand with *n*-hexyl substituents (1),^7^ showed markedly improved solubility in organic solvents.^11^

**Fig. 2:**
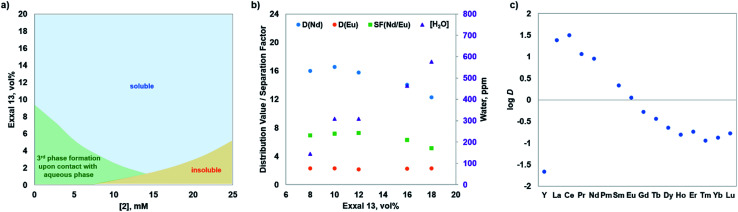
(a) Solubility and phase compatibility diagram of ligand 2 in Isopar L as a function of Exxal 13 volume fraction. (b) Extraction of Nd, Eu, and water from aqueous nitric acid by 2 into organic phase at varying volume fraction of Exxal 13. Organic phase: 4 mM 2 in Isopar L with Exxal 13. Aqueous phase: 1 mM Eu(iii) and 1 mM Nd(iii) in 3 M HNO_3_. (c) Extraction of Ln(iii) (excluding Pm) and Y(iii) with ligand 2. Organic phase: 4 mM 2 in Isopar L with 10 vol% Exxal 13. Aqueous phase: 0.3 mM of each metal in 3 M HNO_3_.

The solubility and third-phase formation compatibility tests were performed to determine the concentration limits of ligand 2 and solvent modifier in the hydrocarbon diluent Isopar L. As can be seen in Fig. 2a, the solubility behaviour follows the expectations of classical solution theory^12^ in that an increase in solubility of 2 is manifested with the addition of solvent modifier Exxal 13 (11-methyl-1-dodecanol with >87 wt% purity). To increase ligand 2 concentration in Isopar L, an addition of higher volume fraction (vol%) of solvent modifier is necessary to attain a homogeneous solution (blue region in Fig. 2a). Although at a lower concentration (5 mM), 2 completely dissolves in Isopar L–Exxal 13 (2.5 vol%) mixture, upon contact with 1 mM Ln(iii) in 3 M nitric acid solution, the organic phase undergoes phase disengagement forming a precipitate (green region in Fig. 2a). The orange region in Fig. 2a represents the incomplete dissolution of 2 in Isopar L–Exxal 13 mixture. The higher concentration of modifier that contains a hydrogen-bond accepting and donating hydroxyl group helps to solubilize both the free ligand and the ligand–metal nitrate complexes in the organic phase. Hence, the increase in volume fraction of modifier results in co-extraction of water, which in turn can influence the separation of Ln(iii). We demonstrate this with Nd(iii) and Eu(iii) extraction using ligand 2 in Isopar L–Exxal 13/nitric acid system. As presented in Fig. 2b, a slight decrease in distribution ratios for Nd(iii) is observed when increasing Exxal 13 concentration above 10 vol% and with it the water concentration in organic phase. Therefore, to prevent the precipitation, while reaching an adequate ligand solvation within the range of ligand concentrations feasible for extraction, and to attain the most efficient separation of f-block metal ions, Isopar L with 10 vol% of Exxal 13 was chosen as the optimal organic medium for the evaluation of ligand 2 complexation with ^241^Am(iii) and Ln(iii) nitrates.

The performance evaluation of ligand 2 in this solvent system revealed its strong preference for both ^241^Am and light Ln(iii). First, a solution of 4 mM ligand 2 in Isopar L with 10 vol% Exxal 13 was contacted with 3 M nitric acid containing 0.1 mM Eu(NO_3_)_3_ spiked with ^241^Am and ^152^Eu radiotracers. The results revealed that both metals are equally well extracted into the organic phase by ligand 2 (*D*_Am_ = 268 ± 13, *D*_Eu_ = 279 ± 14), with no apparent selectivity for one or the other. While this result may seem surprising in the view of the presence of two N-donor atoms in ligand 2, we had shown earlier^7^ that the selectivity between trivalent 4f- and 5f-metals results from an interplay involving structural and electronic-structure effects in the family of bis-amide phenanthroline ligands. In fact, by lowering the electron density on the amide oxygens, by the virtue of incorporating electron-withdrawing groups on the lactam rings, and ultimately increasing the softness of the oxygen donors, excellent selectivity for Am(iii) over Eu(iii) was achieved.^7^

The pre-organization of the four-donor binding site in 2 leads to an efficient differentiation among the 14 lanthanides tested (excluding Pm) and yttrium, with strong preference for metal ions with larger ionic radii. As shown in Fig. 2c, under loading conditions ligand 2 nearly quantitatively extracts La and Ce, showing steady decrease in extraction from Pr to Dy, and limited complexation with Ho–Lu. These results are consistent with ligand 1 performance in 1,2-dichloroethane–nitric acid system, implying that the size of the binding cavity is solely responsible for the observed intra-lanthanide selectivity.^8^ This also highlights the efficiency of this ligand for recovery of Ln, since La alone can comprise up to 30% by mass of all Ln present in used nuclear fuel.^13^ Other neutral extractants, such as TODGA and CMPO show the reversed selectivity trend in the extraction across the trivalent Ln series.^14^

**Fig. 3:**
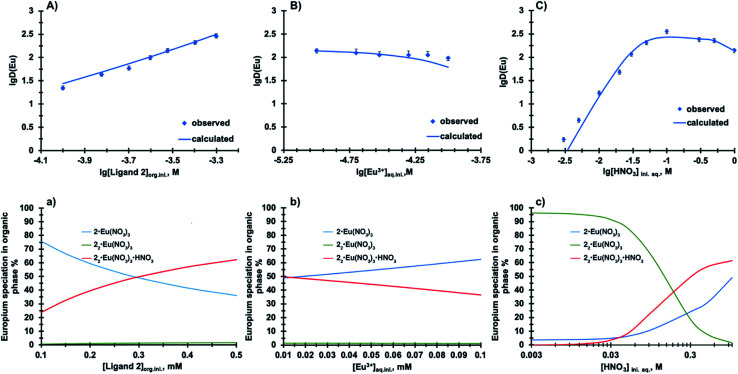
Calculated *versus* experimentally observed distribution data (A, B, C) and speciation diagrams (a, b, c) of Eu(iii) and ligand 2 complexes in the organic phase. (A, a) Organic phase: 0.1–0.5 mM ligand 2 in *n*-dodecane with 10 vol% 1-octanol; aqueous phase: 1 M HNO_3_ and 0.01 mM Eu(NO_3_)_3_. (B, b) Organic phase: 0.3 mM ligand 2 in *n*-dodecane with 10 vol% 1-octanol; aqueous phase: 1 M HNO_3_ and 0.01–0.1 mM Eu(NO_3_)_3_. (C, c) Organic phase: 0.3 mM ligand 2 in *n*-dodecane with 10 vol% 1-octanol; aqueous phase: 0.003–1 M HNO_3_ and 0.01 mM Eu(NO_3_)_3_.

To probe the stoichiometry of the complexes in the organic phase, we chose two metal ions Nd(iii) and Tm(iii) representing early and late lanthanides. The ligand 2 concentration was varied from 0.5 mM to 2 mM at constant 1 mM Ln(iii) loading to cover a range of ligand to metal ratios. The results presented in Fig. SI-7† show that the slope for Nd(iii) and Tm(iii) is 2.02 and 2.07 at 1–2 mM 2, respectively, suggesting a uniform complexation of 2 with lanthanides across the series. To support this finding, the extraction equilibrium analysis was performed based on ^152^Eu distribution ratios summarized in Table SI-4.† These values were obtained from three extraction series where the concentration of components (ligand 2, nitric acid and Eu^3+^) was varied one at a time. Utilizing the distribution ratios as input numbers, the equilibrium constants were modelled for plausible complexes formed during Eu^3+^ extraction using the solvent-extraction modelling program SXLSQI.^15^ The results from SXLSQI model with five chemical equilibria eqn (1)–(5) are compared to the experimental values in Fig. 3(A–C). The comparison between calculated values and experimental data results in agreement factor of 1.93.^16^ From the multiple chemical equilibria involved in the Eu^3+^ extraction, 1 : 1 and 2 : 1 ligand to Eu(NO_3_)_3_ complexes dominate the speciation in the organic phase that is composed of compound 2 in *n*-dodecane with 10 vol% 1-octanol.^17^ Note that the formation of the nitric acid adducts in eqn (1) and (2) is inferred, as acid extraction by 2 was not measured.1
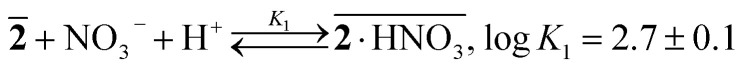
2
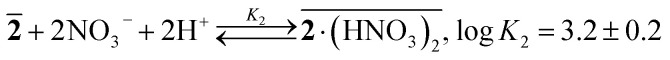
3
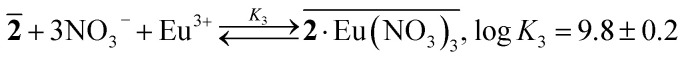
4
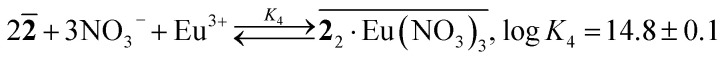
5
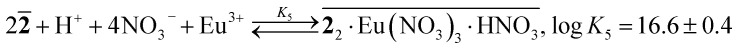


At a low ligand 2 concentration, the 1 : 1 ligand to Eu(NO_3_)_3_ complexes are the predominant species present in the solution (eqn (3), Fig. 3a). Formation of 2 : 1 ligand to Eu(NO_3_)_3_ complexes increases with increase in ligand 2 concentration at constant metal ion loading (eqn (4) and (5)). The reverse trend is observed with increasing Eu(NO_3_)_3_ concentration, where likely one ligand molecule dissociates from the 2 : 1 complex to bind with an incoming metal ion (Fig. 3b). At a low to moderate nitric acid concentrations (Fig. 3c), the 2 : 1 ligand 2 to Eu(NO_3_)_3_ complexes represent the dominant species in the organic phase, but with the increase in aqueous acidity, the speciation changes and is dominated by 1 : 1 and 2 : 1 complexes, as well as 2 : 1 HNO_3_ adduct. These findings are supported by the other phenanthroline-based ligand systems, where the formation of 2 : 1 ligand to metal ion complexes has been elucidated.^6,18^

## Conclusions

The obtained results show that the new bis-lactam-1,10-phenanthroline (BLPhen) ligand efficiently extracts trivalent Am and light Ln from aqueous acidic medium into aliphatic solvent. The analysis comparison of the SXLSQI model with the experimentally measured data confirms the presence of 1 : 1 and 2 : 1 ligand to metal ion complex species in the organic phase of Isopar L–Exxal 13/nitric acid system. Our results present a deeper insight into relations between the performance, functionality and structure of phenanthroline-based ligands, and will guide the rational design of new, tailor-made ligands with desirable properties for f-element extraction and separation, ushering the development of sustainable nuclear fuel cycles.

## Experimental

### Synthesis

To BLPhen precursor (1.0 g, 2.22 mmol) dissolved in dry DMF (22 mL, 0.1 M) was added NaH (0.22 g, 5.55 mmol) under inert atmosphere. After 20 minutes, 2-octyl-1-bromododecane (2.26 mL, 6.66 mmol) was added and the reaction mixture was heated at 85 °C for 12 hours. Afterwards, the reaction mixture was allowed to cool to room temperature, before being diluted with water (∼0.01 M). The formed precipitate was filtered off, washed with water and dried to yield crude product. The product was purified on CombiFlash R_*f*_ automated flash chromatography system using normal phase silica gel as a stationary phase and gradient from 0% to 20% of MeOH in CH_2_Cl_2_ as an eluent system. The product was purified one more time using isocratic 3% of MeOH in CH_2_Cl_2_ as an eluent system. Compound 2 was obtained as brown oil (0.40 g, 19% yield). ^1^H NMR (400 MHz, CDCl_3_) 8.05 (s, 2H), 7.73 (s, 2H), 4.50–4.35 (m, 2H), 3.80–3.72 (m, 2H), 3.55–3.47 (m, 2H), 2.85–2.72 (m, 2H), 2.61–2.54 (m, 2H), 2.37–2.29 (m, 2H), underneath 2.2–2.1 signal (m, 2H), 2.04–1.92 (m, 2H), 178–1.66 (m, 4H), 1.66–1.57 (m, 2H), 1.54–1.46 (m, 2H), 1.4–1.1 (m, 56H), 0.91–0.78 (m, 12H). ^13^C NMR (100.66 MHz, CDCl_3_) 160.6 (CH_0_), 145.8 (CH_0_), 144.1 (CH_0_), 135.9 (CH_1_), 134.5 (CH_0_), 130.2 (CH_0_), 127.2 (CH_1_), 63.7 (CH_1_), 51.0 (CH_2_), 48.9 (CH_1_), 44.7 (CH_1_), 42.3 (CH_1_), 35.3 (CH_1_), 32.0 (CH_2_), 31.8 (CH_2_), 31.6 (CH_2_), 30.3 (CH_2_), 30.2 (CH_2_), 29.8 (CH_2_), 29.5 (CH_2_), 29.2 (CH_2_), 27.7 (CH_2_), 26.6 (CH_2_), 22.8 (CH_2_), 14.3 (CH_3_). Additionally, the proton and carbon assignments were verified with COSY, HMQC and NOESY NMR experiments. HRMS *m*/*z* [M + H]^+^ and [2M + K + H]^2+^, calculated for C_64_H_98_N_4_O_2_, 955.7763 and C_128_H_197_KN_8_O_4_, 974.75420; found 955.7758 and 974.75080, respectively.

### Solvent extraction experiments

(A) All experimental work with ^241^Am was conducted in radiological facility. A 500 microliter (μL) aqueous phase consisting of 0.1 mM Eu(NO_3_)_3_ in 3 M HNO_3_-spiked with 5 μL of 1.85 × 10^3^ kBq each ^241^Am and ^152^Eu-was contacted with an equal volume of organic phase containing 1.0 mM of ligand 2 in Isopar L with 10 vol% Exxal 13. The two phases were contacted at a 1 : 1 ratio of organic/aqueous by end-over-end rotation in individual 1.8 mL capacity snap-top Eppendorf tubes using a rotating wheel in an air box set at 25.5 °C ± 0.5 °C. Contacts were performed in duplicate using contact time of 1 hour. Following contacting, the duplicate samples were subjected to centrifugation at 1811 × *g* for five minutes at 25 °C to separate the phases. Each phase was then subsampled, with 250 μL volumes isolated from each phase and transferred to polypropylene tubes for counting using a Canberra Gamma Analyst Integrated Gamma Spectrometer.

(B) Equal volumes of aqueous and organic phases, 750 microliter (μL) each, were contacted for 1 hour in individual 1.8 mL capacity snap-top Eppendorf tubes using a rotating wheel set at 60 rpm and placed in an air box set at 25 °C ± 0.5 °C. Following contacting, the samples were subjected to centrifugation for 5 minutes at 1811 × *g*. Then, the samples were subsampled by removing 600 μL of the organic phase, which was subjected to Karl Fischer titration. The interface layer, if any, was removed using a plastic pipette, prior to subsampling the lower aqueous phase. The remaining aqueous layer was then analyzed by ICP-OES and/or IC to determine the metal and nitrate concentrations in the organic phase. All extractions were performed in duplicate.

## Associated content

Experimental and modeling details and NMR data (PDF).

## Conflicts of interest

The authors declare no competing financial interests.

## Supplementary Material

RA-009-C9RA06115K-s001
