# Supplementary material for: Sequestration of trivalent americium and lanthanide nitrates with bis-lactam-1,10-phenanthroline ligand in a hydrocarbon solvent
Source: RSC Adv. 2019 Aug 23;9(46):26537–41. doi: 10.1039/c9ra06115k (PMC9070534; doi:10.1039/c9ra06115k)
Supplement: RA-009-C9RA06115K-s001 [file RA-009-C9RA06115K-s001.pdf]

Electronic Supplementary Material (ESI) for RSC Advances.
This journal is © The Royal Society of Chemistry 2019

SUPPORTING INFORMATION

Sequestration of Trivalent Americium and Lanthanide Nitrates with Bis-Lactam-1,10-Phenanthroline Ligand in a Hydrocarbon Solvent

Yana Karslyan[a,b], Frederick V. Sloop[b], Laetitia Delmau[c], Bruce A. Moyer[b], Ilja Popovs,[b] Alena Paulenova[a], and Santa Jansone-Popova[b*]

[a] *Laboratory of Transuranic Elements, Department of Chemistry, Oregon State University, Corvallis, OR, 97331*

[b] *Chemical Sciences Division, Oak Ridge National Laboratory, Oak Ridge, TN 37831*

[c] *Isotope and Fuel Cycle Technology Division, Oak Ridge National Laboratory, Oak Ridge, TN, 37831*

Table of Contents

Materials and Methods **2**

Synthesis **2**

Solubility and Phase Compatibility Experiments **6**

Solvent Extraction Experiments **6**

Inductively Coupled Plasma Optical Emission Spectroscopy **7**

Gamma Spectroscopy **7**

Karl Fischer Titration **8**

Ion Chromatography **8**

Extraction Equilibrium Analysis **10**

References **11**

Materials and Methods

The 1D and 2D spectra were recorded on an AvanceIII-400 MHz NMR spectrometer (Bruker Company) equipped with a 5 mm PABBO probe. For ^{1}H and ^{13}C NMR spectra, residual solvent peak was used as an internal reference ($CDCl_3$: 7.25 ppm for ^{1}H NMR and 77.16 ppm for ^{13}C NMR). For ^{13}C NMR, multiplicities were distinguished using DEPT experiments. Proton assignments were verified with COSY and NOESY experiments as appropriate. HRMS analyses were performed under contract by UT Austin mass spectrometric facility using positive mode ESI method. Commercially available compounds were purchased from Aldrich Chemical Co., Acros Organics, Alfa Aesar or TCI America and were used without further purification. Isopar L, a mixture of C6 through C13 hydrocarbons, was used as a diluent. Exxal 13, 98 w% of iso-tridecyl alcohol, was used as a solvent modifier. Both the diluent and modifier were used as received, without further purification. The stock solutions of ligands were prepared by weighing a desired amount of a ligand and adjusting the diluent and solvent modifier volumes depending on the experiment series. The aqueous solutions of trivalent lanthanides, Ln(III), were prepared using the single element 10,000 ppm standards (High-Purity Standards). The nitric acid (ULTREX II from J.T. Baker) was added to adjust the concentration of Ln(III) in the aqueous phases. The resultant acid concentrations were determined by titration with standardized 0.1 M KOH solutions (Fisher Chemical) and phenolphthalein. All aqueous solutions were prepared and diluted to the desired concentrations and volumes with deionized water (Milli-Q, 18.2MΩ-cm).

Synthesis

The starting material **A** (*(4aS,8bS,12aS,18aS)-1,2,3,4,4a,8b,9,10,11,12,12a,13,18,18a-tetradecahydro-1,4:9,12-dimethanodiquinolino[3,4-b:4',3'-j][1,10]phenanthroline-14,17-dione*) was synthesized in 5 steps from *2,9-dimethyl-1,10-phenanthroline* according to a literature procedure (see reference 7 in main text).

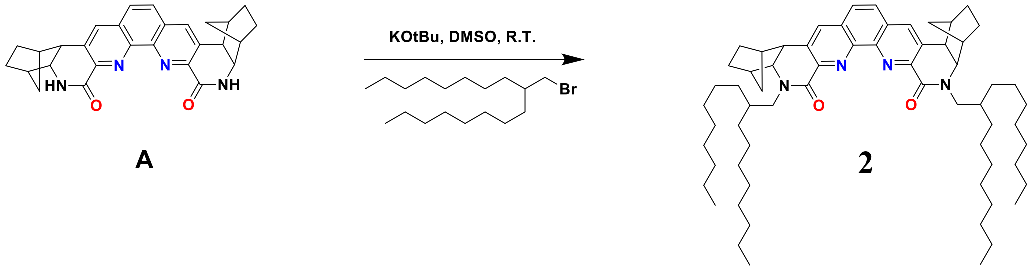

*(4aS,8bS,12aS,18aS)-13,18-bis(2-octyldecyl)-1,2,3,4,4a,8b,9,10,11,12,12a,13,18,18a-tetradecahydro-1,4:9,12-dimethanodiquinolino[3,4-b:4',3'-j][1,10]phenanthroline-14,17-dione (**2**).* To compound **A** (1.0 g, 2.22 mmol) dissolved in dry DMF (22 mL, 0.1 M) was added NaH (0.22 g, 5.55 mmol) under inert atmosphere. After 20 minutes, 2-octyl-1-bromododecane (2.26 mL, 6.66 mmol) was added and the reaction mixture was heated at 85 °C for 12 hours. Afterwards, the reaction mixture was allowed to cool to room temperature, before being diluted with water (~0.01 M). The formed precipitate was filtered off, washed with water and dried to yield crude product. The product was purified on CombiFlash R_f automated flash chromatography system using normal phase silica gel as a stationary phase and gradient from 0% to 20% of MeOH in CH_2Cl_2 as an eluent system. The product was purified one more time using isocratic 3% of MeOH in CH_2Cl_2 as an eluent system. The product was obtained as brown oil (0.40 g, 19% yield). **^{1}H**

NMR (400 MHz, $CDCl_3$) 8.05 (s, 2H), 7.73 (s, 2H), 4.50-4.35 (m, 2H), 3.80-3.72 (m, 2H), 3.55-3.47 (m, 2H), 2.85-2.72 (m, 2H), 2.61-2.54 (m, 2H), 2.37-2.29 (m, 2H), underneath 2.2-2.1 signal (m, 2H), 2.04-1.92 (m, 2H), 178-1.66 (m, 4H), 1.66-1.57 (m, 2H), 1.54-1.46 (m, 2H), 1.4-1.1 (m, 56H), 0.91-0.78 (m, 12H). ^{13}**C NMR** (100.66 MHz, $CDCl_3$) 160.6 (CH_0), 145.8 (CH_0), 144.1 (CH_0), 135.9 (CH_1), 134.5 (CH_0), 130.2 (CH_0), 127.2 (CH_1), 63.7 (CH_1), 51.0 (CH_2), 48.9 (CH_1), 44.7 (CH_1), 42.3 (CH_1), 35.3 (CH_1), 32.0 (CH_2), 31.8 (CH_2), 31.6 (CH_2), 30.3 (CH_2), 30.2 (CH_2), 29.8 (CH_2), 29.5 (CH_2), 29.2 (CH_2), 27.7 (CH_2), 26.6 (CH_2), 22.8 (CH_2), 14.3 (CH_3). Additionally, the proton and carbon assignments were verified with COSY, HMQC and NOESY NMR experiments. **HRMS** *m/z*: $[M+H]^+$ and $[2M+K+H]^{2+}$, calculated for $C_{64}H_{98}N_4O_2$, 955.7763 and $C_{128}H_{197}KN_8O_4$, 974.75420; found 955.7758 and 974.75080, respectively.

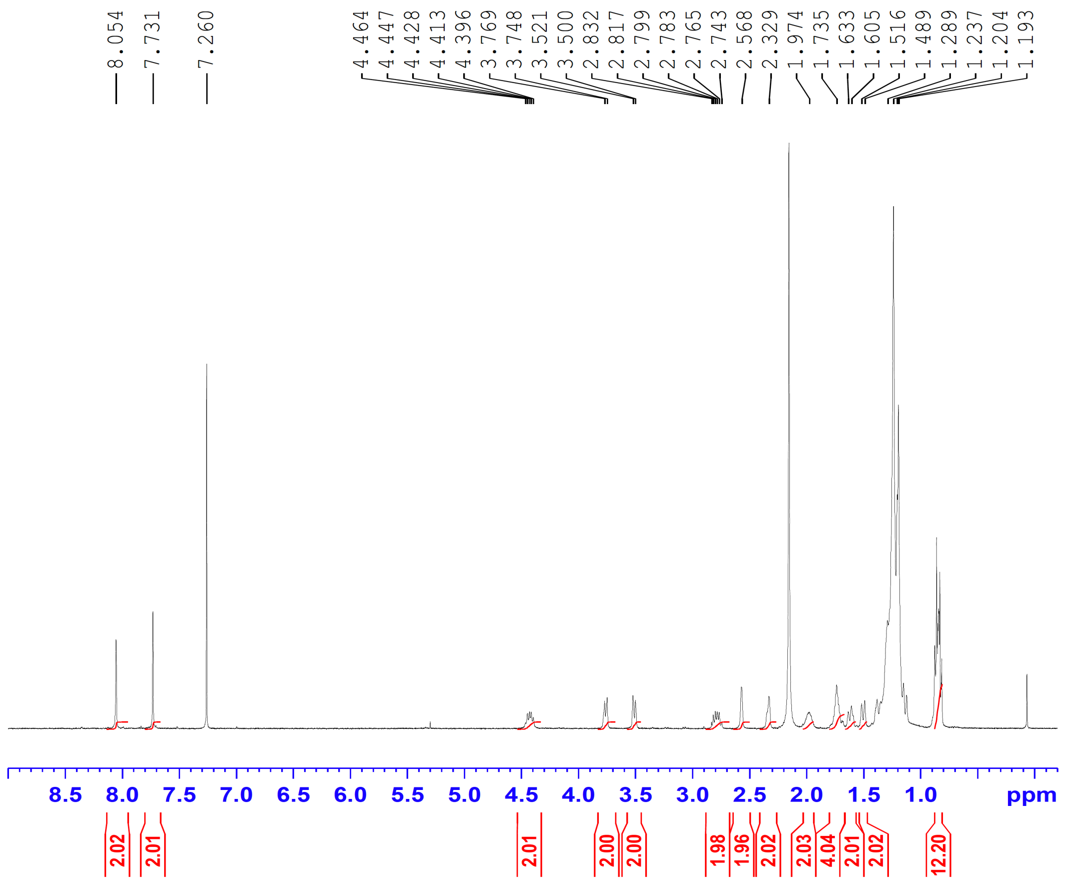

Figure SI-1. ^{1}H NMR spectrum.

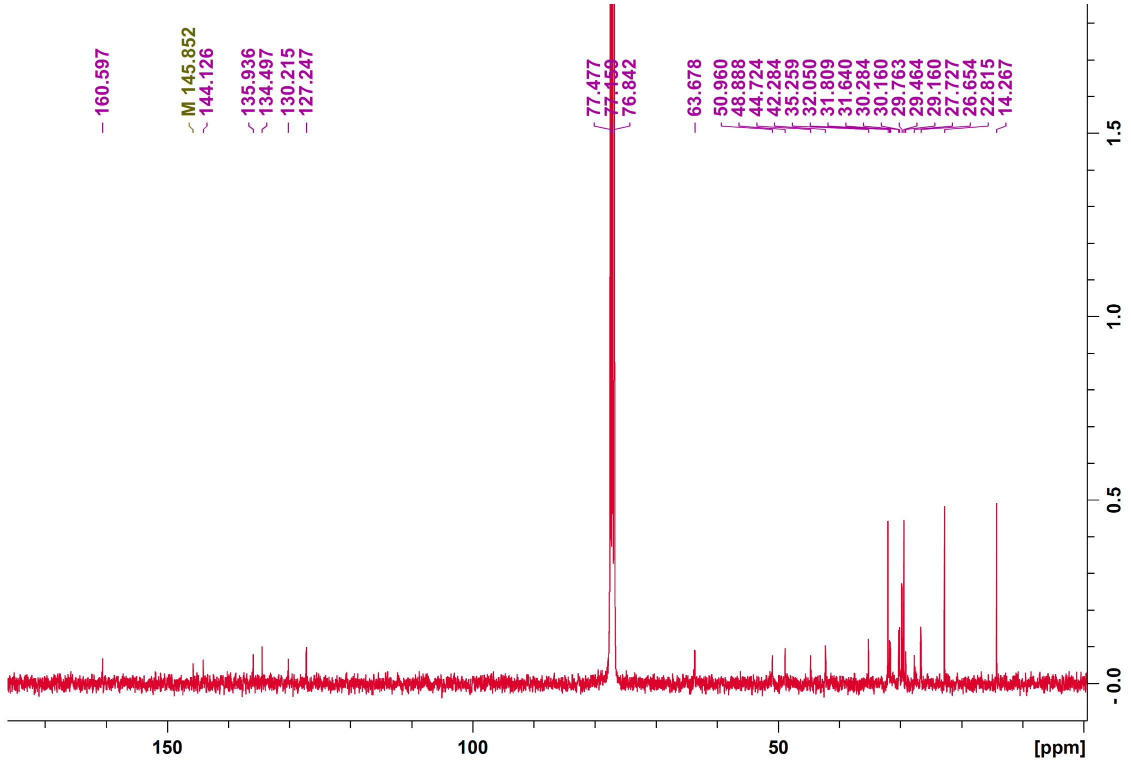

Figure SI-2. ^{13}C NMR spectrum.

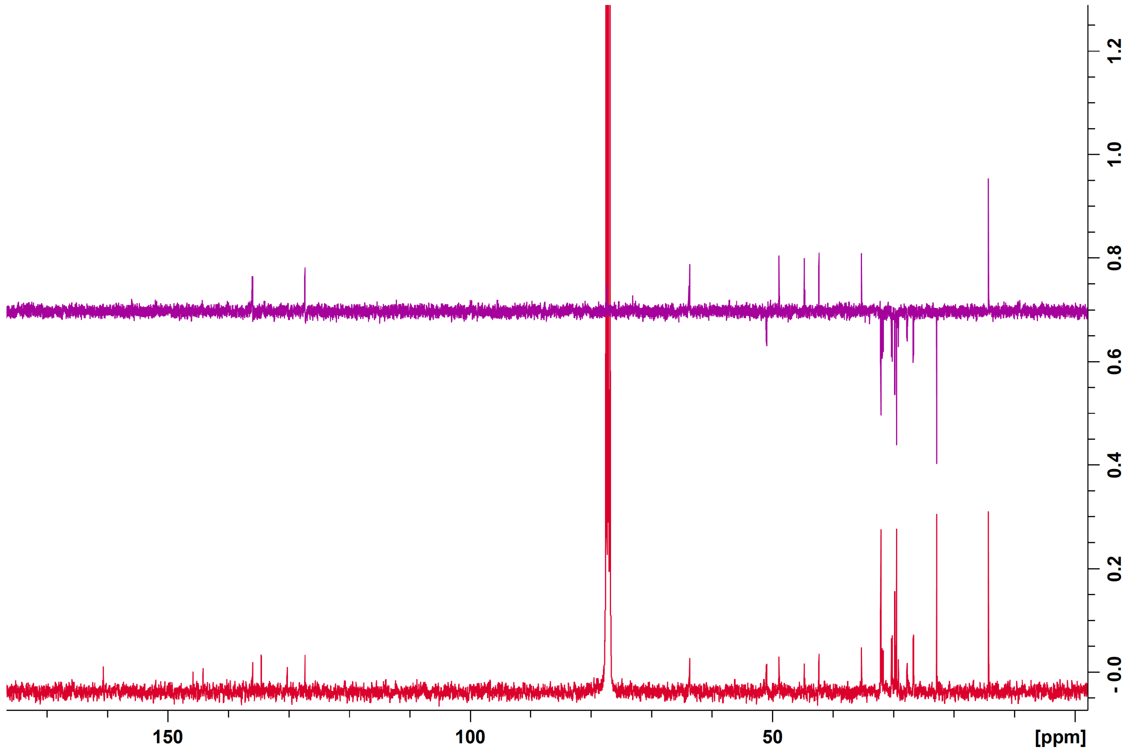

Figure SI-3. Overlay of ^{13}C and DEPT NMR spectra.

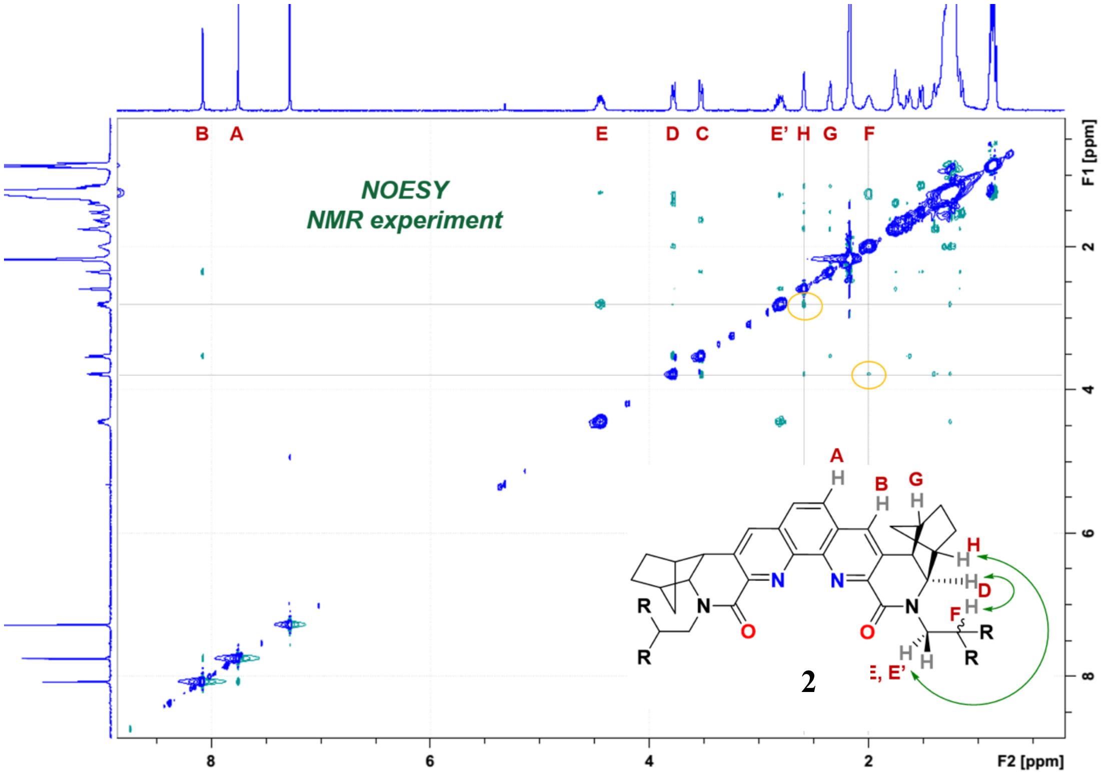

Figure SI-4. ^1H-^1H NOESY NMR spectrum with proton-proton through space correlation assignments.

The compound **B** was synthesized according to a literature procedure (see reference 7 in main text).

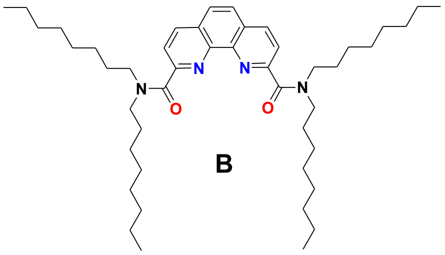

B

Solubility and Phase Compatibility Experiments

The solubility of ligands was evaluated by dissolving a weighed amount of each compound in Isopar L and Isopar L - Exxal 13 mixtures. The ultrasonic bath was used as a homogenizer. The formation or absence of third phase was observed when organic phase samples were contacted with equal volumes of 0.1 mM Eu(III) nitrate in 3 M HNO_3.

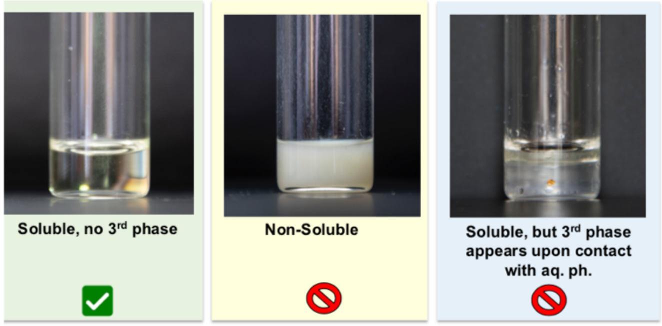

a) b) c)

Figure SI-5. Examples of organic phases before and after contact with aqueous phase: a) 15 mM **1** in Isopar with 1.5 vol % Exxal 13, b) 4.8 mM **1** in Isopar L with 5 vol % Exxal 13, contacted with 0.1 mM Eu(III) in 3 M HNO_3, c) 4 mM **1** in Isopar L with 10 vol % Exxal 13, contacted with 0.1 mM Eu(III) in 3 M HNO_3.

Solvent Extraction Experiments

A) *Caution: care should be exercised when manipulating radiolabeled solutions by wearing proper PPE, utilizing appropriate engineering controls, and applicable waste streams. All experimental work with ^{241}Am was conducted in radiological facility.* A 500 microliter (µL) aqueous phase consisting of 0.1 mM $Eu(NO_3)_3$ in 3 M HNO_3–spiked with 5 µL of 1.85×10^3 kBq each ^{241}Am and ^{152}Eu–was contacted with an equal volume of organic phase containing 1.0 mM of ligands **2** and **B** in Isopar L with 10 vol% Exxal 13. The two phases were contacted at a 1:1 ratio of organic/aqueous by end-over-end rotation in individual 1.8 mL capacity snap-top Eppendorf tubes using a rotating wheel in an air box set at 25.5 °C ± 0.5 °C. Contacts were performed in duplicate using contact time of 1 hour. Following contacting, the duplicate samples were subjected to centrifugation at 1,811 × g for five minutes at 25 °C to separate the phases. Each phase was then subsampled, with 250 µL volumes isolated from each phase and transferred to polypropylene tubes for counting using a Canberra Gamma Analyst Integrated Gamma Spectrometer.

B) Equal volumes of aqueous and organic phases, 750 microliter (µL) each, were contacted for 1 hour in individual 1.8 mL capacity snap-top Eppendorf tubes using a rotating wheel set at 60 rpm and placed in an air box set at 25 °C ± 0.5°C. Following contacting, the samples were subjected to centrifugation for 5 mins at 1,811 × g. Then, the samples were subsampled by removing 600 µL of the organic phase, which was subjected to Karl Fisher titration. The interface layer, if any, was removed using a plastic pipette, prior to subsampling the lower aqueous phase. The remaining aqueous layer was then analyzed by ICP-OES and/or IC to determine the metal and nitrate concentrations in the organic phase. All extractions were performed in duplicate. The organic phases where not pre-equilibrated with acidic, metal-free aqueous phases, since it was expected

that this might have an insignificant effect on the accuracy compared to volumetric transfers, sampling, laboratory temperature variation etc.

The values of metal distribution ratio, D, were obtained using Eq.1:

$$D(metal) = \frac{[M]_{org}}{[M]_{aq}} = \frac{[M]_{aq,ini} - [M]_{aq}}{[M]_{aq}} \qquad \text{Eq. 1}$$

where $[M]_{org}$ = metal concentration or counts rate (for radiolabeled samples) in organic phase after extraction, $[M]_{aq.ini}$ and $[M]_{aq}$ = metal concentration or count rate (for radiolabeled samples) in aqueous phase before and after extraction, respectively.

Separation factor (*SF*) values were calculated according to:

$$SF_{m1/m2} = \frac{D(metal\ 1)}{D(metal\ 2)} \qquad \text{Eq. 2}$$

Errors for D Values are calculated according to:

$$\frac{\sigma_D}{D} = \frac{\frac{C_{in}}{C_{aq}^2}\sigma_{aq}}{D} \qquad \text{Eq. 3}$$

where C_{in} and C_{aq} are the aqueous metal concentrations before and after extraction, respectively, and σ_{aq} is the calculated standard deviation of the measured C_{aq}.
Error bars for Log D values are calculated according to:
Where A = Log D

$$\frac{dA}{dT} = \frac{\log e}{D} \qquad \text{Eq. 5}$$

$$\sigma_A = \sigma_D \times \frac{0.434}{D} \qquad \text{Eq. 6}$$

The overall accuracy of the employed technique falls in the 5-10 % range.

Inductively Coupled Plasma Optical Emission Spectroscopy

Each aqueous phase was subsampled, with 100-500 µL aliquots of the aqueous phases transferred to individual 15 mL polypropylene tubes containing 2.4-2.5 mL of 4% HNO_3 for analysis using ICP-OES (Thermo Scientific iCAP 7400 ICP-OES Duo). Two samples of the initial aqueous phases (i.e., prior to extraction) were also prepared for the analysis. Calibration curves covered 0.5-20 ppm metal concentration range with R^2 value for linear trendline of 1. The areas found under the observed peaks were used to determine the metal concentration in the contacted solutions.

Gamma Spectroscopy

250 µL of aqueous and 250 µL of organic phases were subsampled after extraction described earlier and analyzed by gamma-spectrometry using a Canberra Gamma Analyst Integrated Gamma Spectrometer fitted with a HPGe detector, U-type cryostat and integrated Dewar, designed to operate in the 50 keV to >10 MeV range. The peaks considered were those at 59.5 keV for ^{241}Am and 121.8 keV for ^{152}Eu. The areas found under those respective peaks were used for determining distribution (D) values. The counting times were dependent on the activity found in the respective

phases. Often the measurement times were set to 60 mins at the beginning, then in order to obtain a relative standard deviation of the counting statistics lower than 1%.

Karl Fischer Titration

The partitioning of water in organic phase was analyzed under varying Exxal 13 concentrations in Isopar L. The 600 μL sample aliquots of organic phases were analyzed by Metrohm 310 Karl Fisher Coulometer. HYDRANAL-Coulomat AG (Fluka) was used as a reagent for analysis. The injection by syringe, without dipping the needle, was used to deliver subsamples to the titrator. The validation of instrument was performed using HYDRANAL Water Standard 0.1 (0.10 ± 0.005 mg/g) and accuracy of KF analysis was found to be 0.11 ± 0.010 mg/g. The accuracy of analysis was checked periodically during the experiment. The experimental error was estimated by Eq.3 using at least duplicate samples. The experimentally determined water partitioning into ligand-free solvent was determined to be 14 mg/g and was accounted for during analysis.

Ion Chromatography

The nitrate quantification was performed by ion chromatograph Dionex ICS-5000+ Reagent-Free High-Pressure, which contains a built-in conductivity detector. The instrument was equipped with Thermo Scientific Dionex IonPack AS11-HC Hydroxide-Selective Anion-Exchange Column (4x250 mm) and IonPack AG11-HC Guard Column (4x50 mm). Solution of 30 mM KOH was used as an eluent. The flow rate of 1.2 mL/min was set at 112 mA conductivity. Analyte chromatograms were plotted and data handled using a Chromeleon software (version 6.2). The calibration curve was prepared by diluting 1000 ppm nitrate IC standard (Inorganic Ventures) to a series of concentrations containing 5, 10, 25, 50, 75, 100 and 125 ppm of nitrate. The samples of aqueous phase prior and after contact with organic phases were probed by following 250-fold dilution performed volumetrically in two steps. The distribution values were calculated according to the Eq.1, where the measured nitrate concentration was substituted instead of concentration of metal.

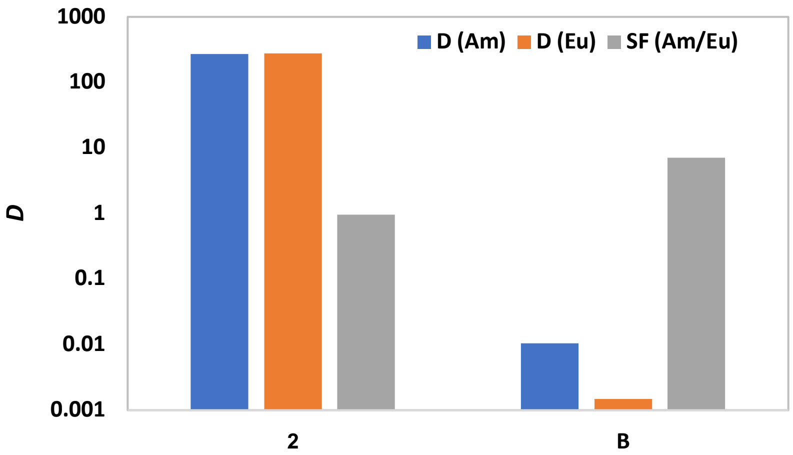

Figure SI-6. Extraction of ^{241}Am and ^{152}Eu from 3 M HNO_3 by ligands **2** and **B** into Isopar L with 10 vol% Exxal 13. Organic phase: 4 mM ligand.

Table SI-1. Trans-lanthanide separation factors using ligand **2**. Organic phase: 4 mM ligand **2** in Isopar L with 10 vol % Exxal 13. Aqueous phase: 0.3 mM of each Ln (except Pm) and Y in 3 M HNO_3.

	La	Ce	Pr	Nd	Sm	Eu	Gd	Tb	Dy	Ho	Er	Tm	Yb	Lu
Y	9.0E-04	6.9E-04	1.9E-03	2.4E-03	1.0E-02	1.9E-02	4.1E-02	6.0E-02	9.6E-02	1.4E-01	1.2E-01	1.9E-01	1.6E-01	1.3E-01
La		0.76	2.1	2.7	11.2	21.4	45.6	66.5	106	154	132	213	181	144
Ce			2.7	3.5	14.7	28.0	59.7	87.0	139	202	173	279	237	188
Pr				1.3	5.4	10.3	21.9	31.9	51.1	74.2	63.4	102	86.8	69.0
Nd					4.2	7.9	16.9	24.6	39.4	57.3	48.9	78.9	67.0	53.2
Sm						1.9	4.1	5.9	9.5	13.8	11.8	19.0	16.1	12.8
Eu							2.1	3.1	5.0	7.2	6.2	9.9	8.4	6.7
Gd								1.5	2.3	3.4	2.9	4.7	4.0	3.1
Tb									1.6	2.3	2.0	3.2	2.7	2.2
Dy										1.5	1.2	2.0	1.7	1.3
Ho											0.9	1.4	1.2	0.9
Er												1.6	1.4	1.1
Tm													0.85	0.67
Yb														0.79

Table SI-2. Distribution of Nd(III) and its separation from Eu(III) by **2** in Isopar L as a function of HNO_3 concentration in the aqueous phase. Organic phase: 4 mM **2** ligand in Isopar L with 10 vol % Exxal 13. Aqueous phase: 1 mM Eu(III) and 1 mM Nd(III) in 0.3-5 M HNO_3.

$[HNO_3]_{ini.aq}$, M	D(Nd)	D(Eu)	SF(Nd/Eu)
0.1	12.7±0.6	4.33±0.2	2.93±0.2
1	28.9±1.4	4.43±0.2	6.53±0.3
2	27.2±1.4	4.39±0.2	6.18±0.3
3	20.5±1.0	4.81±0.2	4.25±0.2
5	16.6±0.8	2.31±0.1	7.19±0.4

Table SI-3. Extraction of water and nitric acid into organic phase as a function of varying EXXAL 13 volume fraction. Organic phase: 4 mM **2** in Isopar L with EXXAL 13. Aqueous phase: 1 mM Eu(III) and 1 mM Nd(III) in 3 M HNO_3.

EXXAL 13, v %	H_2O in org.ph., ppm	$D(HNO_3)$
8	145	0.129
10	309	0.120
12	309	0.104
16	464	0.118
18	576	0.103

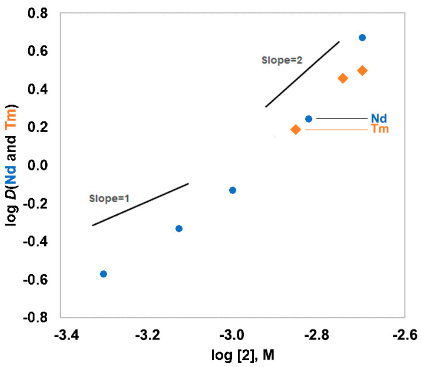

Figure SI-7. Distribution ratios of Nd and Tm as a function of ligand **2** concentration at constant metal and nitric acid initial aqueous concentrations. Organic Phase: 0.5–2 mM **2** in Isopar L with 10 vol% Exxal 13. Aqueous phase: 1 mM Nd(III) and Tm(III) in 3 M HNO_3.

Extraction Equilibrium Analysis

To explore the stoichiometric basis of liquid-liquid distribution phenomena, the modeling via SXLSQI software of europium distribution data was employed. The software allows the user to hypothesize the multiple extraction equilibria between reactant species, the corresponding thermodynamic equilibrium constants (log *K* values), the stoichiometry of product species, and, eventually, compare the calculated vs. observed distribution data. The calculated distribution values are defined based on the concentrations of all of the species at equilibrium, which are, in turn, found from the combination of information provided by user. Among user-supplied information are initial concentrations of all components in the system, experimental D_{Eu} values (Table SI-3), its uncertainties, as well as such adjustable parameters as log *K* values, solubility parameters, molecular volume etc. needed for the activity-coefficient calculations, phase-volume changes and concentration-scale interconversions. The user supplied logarithms of *K* serve as the initial estimates for the program. The thermodynamic equilibrium constants are defined as activity quotients in the standard mass-action formalism:

$$K_1 = \frac{[\mathrm{L{\cdot}Eu(NO_3)_3}]_{\mathrm{org}}\, g_{\mathrm{L{\cdot}Eu(NO_3)_3}}}{[\mathrm{Eu^{3+}}]_{\mathrm{aq}}\, g_{\mathrm{Eu^{3+}}} [\mathrm{NO_3^-}]_{\mathrm{aq}}\, g^3_{\mathrm{NO_3^-}}\, [\mathrm{L}]_{\mathrm{org}}\, g_{\mathrm{L}}} \qquad \text{Eq. 7}$$

where *g* stands for the activity coefficient defined on the molarity scale and estimated by SXLSQI using the Pitzer[i] and Hildebrand-Scott[ii] treatment for organic-phase and aqueous-phase activity coefficients, respectively.

The *reactant species* (organic-phase extractant molecules **2** and aqueous ions H^+, NO_3^-, Eu^{3+}) and the *product species* (extraction complexes in the organic phase, see main text, Eq.1-4) are being confirmed by simultaneous solution of the equilibrium-quotient expressions for each hypothesized equilibrium and the conservation-of-mass expressions for each component of system in the equilibria.

The accuracy of fit using user-supplied data and modeled data is defined via the least-square regression analysis and is reported as an agreement factor, which is a weighed root-mean-square deviation of the calculated vs. observed D_{Eu}.[6] The closer agreement factor to 1, better is the fit. When uncertainty of fitting is equal to that of distribution values, an agreement factor approaches

unity. For fitting to be unbiased, 20 refinement cycles were performed for each final converged estimate of log K values and the minimum agreement factor was reached within user-defined model.

The goodness of the fit for user-supplied and modeled data is defined using the least-square regression analysis and is reported as an agreement factor, which is a weighed root-mean-square deviation of the calculated vs. observed D(Eu):

$$\sigma = \left[\sum w_i (Y_i - Y_{c,i})^2 / (N_o - N_p)\right]^{1/2},$$

where Y_i is the experimentally observed quantity (i.e., log $D(Eu)$), $Y_{c,i}$ is the corresponding quantity calculated from the model being tested, w_i is the weighting factor defined as the reciprocal of the square of the estimated uncertainty of Y_i, N_o is the number of observations, and N_p is the number of adjustable parameters (i.e., log$K$ values). Given how the weighting is defined, the value of σ will approach unity when the error of fitting is equal to the estimated experimental error. Ideally, a perfect fit should yield a value of unity.

Table SI-4. Summary of Eu(III) extraction results. Variation of ligand **2** concentration at constant 0.01 mM $Eu(NO_3)_3$ and 1 M HNO_3. Variation of nitric acid concentration at constant 0.01 mM $Eu(NO_3)_3$ and 0.3 mM ligand **2**. Variation of Eu(III) concentration at constant 0.3 mM ligand **2** and 1 M HNO_3. Organic phase medium is *n*-dodecane with 10 vol% 1-octanol.

Organic-phase Ligand Variation, mM	D(Eu)	D(Eu) Uncertainty	Aqueous-phase Acid Variation, M	D(Eu)	D(Eu) Uncertainty	Aqueous-phase Metal Loading Variation, mM	D(Eu)	D(Eu) Uncertainty
0.1	22	2	0.003	1.73	0.4	0.01	140	14
0.15	43	4	0.005	4.5	0.5	0.02	125	14
0.2	59	10	0.01	17.2	1.7	0.03	112	17
0.25	99	13	0.02	48.2	4.8	0.05	112	11
0.3	140	14	0.03	117	45	0.07	112	11
0.4	209	45	0.05	208	21	0.1	96	9.6
0.5	289	73	0.1	356	36			
			0.3	240	16			
			0.5	230	77			
			1.0	140	14			

Table SI-5. Summary of input parameters used in SXLSQI modeling (25 °C)

Species or Interaction	$\beta^{(0)}$, (kg/mol)$^{1/2}$	$\beta^{(1)}$, (kg/mol)$^{1/2}$	C^{Φ}, (kg/mol)$^{1/2}$	α_1, (kg/mol)$^{1/2}$	Ref.
HNO_3	0.11190	0.3686	0.0024070	2.0	1
$H^+{-}NO_3$	0.1168	0.3546	-0.00539	2.0	2
$Eu(NO_3)_3$	0.475	5.13	-0.84375	2.0	1

SXLSQI Symbol	Species	Molar wt [1]	Molar volume (cm^3 mol^{-1}) [1]	Dielectric constant [2]
Org. Solv.	Organic Solvent (90% n-dodecane, 10% 1-octanol)	164.82	218.93	2.107
B	Neutral ligand **1**	955.51	672.6	-

SXLSQI Symbol	Species	Molar wt	Molar volume (cm^3 mol^{-1})[3]	V_0 (cm^3 mol^{-1})[4]	S_v (cm^3 l$^{1/2}$ mol$^{-3/2}$)[4]
M	Eu^{3+}	151.86	21.5	21.40	
X	NO_3^-	62.005	29.0	29.33	0.543
H	H^+	1.0079	0.0	0.00	0.000

[1] Molar volumes for organic species were estimated from group parameters from Ref. 3a. N-dodecane molar volume was taken from Ref. 3b. and was equal to 228.6 cm^3 mol^{-1}. 1-octanol molar volume was calculated to be 158.37. Both molar weight and volume of org. solv. were adjusted using the molar fraction of each solvent component.
[2] A combined form of the Jouyban−Acree model and the Abraham solvation parameters from Ref. 3c were used for predicting the dielectric constant of binary organic solvent at 25^{0}C. Dielectric constants of pure n-dodecane and 1-octanol at 20ºC were taken from Ref. 4
[3] Molar volumes for inorganic ions were taken to be approximately equal to V_0.
[4] Masson coefficients account for the small volume changes of the aqueous phase as electrolyte concentrations vary. Values taken from Ref. 5

References

1. Pitzer, K.S. Ion Interaction Approach: Theory and Data Correlation. In Activity Coefficients in Electrolyte Solutions; Pitzer, K.S., Ed.; CRC Press: Boca Raton, FL, 1991; pp 75-153.

2. Campbell, E. L; Holfeltz, V.E.; Hall, G. B.; Nash, K. L; Lumetta, G. J.; Levitskaia, T. G., Nitric Acid and Water Extraction by T2EHDGA in n-Dodecane, Solvent Extraction and ion Exchange, 2017, **35**, 1–18.

3.a) Barton. A.F. Handbook of Solubility Parameters and Other Cohesion Parameters,CRC Press: Boca Raton. FL. 1991. b) Riddick J. A.; Bunger, W. B.; Sakano, T.K., Organic Solvents: Physical Properties and Methods of Purification, Vol. II, Wiley-Interscience: New York, 1986. c) Jouyban, A.; Soltanpour, S., Prediction of Dielectric Constants of Binary Solvents at Various Temperatures, Journal of Chemical & Engineering Data, 2010, **55**, 2951–2963.

4. Maryott, A. A.; Smith, E. R., Table of Dielectric Constants of Pure Liquids, National Burea of Standards, 1951.

5. Millero, F. J. The Partial Molal Volumes of Electrolytes in Aqueous Solutions. In Water and Aqueous Solutions: Structure, Thermodynamics and Transport Processes; Horne. R.A.. Eds.; Wiley: New York, 1972; 519-595.

6. Huber-Carol, C.; Balakrishnan, N.; Nikulin, M. S.; Mesbah, M., eds., *Goodness-of-Fit Tests and Model Validity*, Springer: 2002.
